# Women empowerment and childhood stunting: evidence from rural northwest Ethiopia

**DOI:** 10.1186/s12887-023-04500-5

**Published:** 2024-01-09

**Authors:** Emebet Gashaw Wassie, Mesfin Wogayehu Tenagashaw, Tenaw Yimer Tiruye

**Affiliations:** 1 College of Health Sciences, Debre Markos University, Debre Markos, Ethiopia; 2 Applied Human Nutrition Department, Faculty of Chemical and Food Engineering, Bahir Dar University, Bahir Dar, Amhara Region, Ethiopia; 3 School of Public Health, Debre Markos University, Debre Markos, Ethiopia; 4 Allied Health and Human Performance, University of South Australia, Adelaide, Australia

**Keywords:** Women empowerment, Stunting, Nutrition, Ethiopia

## Abstract

**Background:**

Women are often the primary caregivers of children, and as such, their empowerment could influence the nutritional status of their children. However, the role of maternal empowerment on the nutritional status of children in Ethiopia is largely unknown.

**Aim:**

To determine the association of women’s empowerment with childhood stunting in rural northwest Ethiopia.

**Methods:**

A community-based cross-sectional study was conducted among 582 mothers with children aged 6–59 months. A multistage sampling technique was used to select the study participants. Binomial logistic regression analyses were used to assess whether women’s empowerment (categorized as low, moderate and high) and its five dimensions (household decision-making, educational status, cash earnings, house/land ownership, and membership in community groups) were associated with stunting in children. Odds ratios with 95% CI were estimated, and statistical significance was declared at a p-value of < 0.05.

**Results:**

A total of 114 (19.6%), 312 (53.6%), and 156 (26.8%) participants had low, moderate, and high empowerment levels, respectively. In addition, 255 (43.8%) mothers had children who were stunted (too short for their age). In the adjusted models, mothers with moderate empowerment (AOR 0.60, 95% CI: 0.35, 0.97) and high empowerment (AOR 0.56, 95% CI: 0.37, 0.86) had lower odds of having stunted children compared to mothers with low empowerment. Mothers who had a secondary education or higher (AOR 0.57, 95% CI: 0.35, 0.93), owned a house or land (AOR 0.64, 95% CI: 0.44, 0.94) and were members of community groups (AOR 0.54, 95% CI: 0.36, 0.80) were less likely to have stunted children.

**Conclusion:**

High women empowerment was significantly associated with a lower likelihood of childhood stunting. The findings suggest a need to look beyond the direct causes of stunting and incorporate targeted strategies for empowering women into child nutrition programs.

## Background

The World Health Organisation (WHO) defines malnutrition as “deficiencies or excesses in nutrient intake, imbalance of essential nutrients or impaired nutrient utilization” [1]. Stunting is a form of undernutrition which is defined as a height-for-age z-score more than two standard deviations below the age- and sex-specific WHO Child Growth Standards reference median [2]. Globally, about 155 million children under 5 years of age are stunted [3]. Africa was the only region where the number of stunted children increased from 50.6 million to 58.7 million between 2000 and 2017 [4]. Ethiopia is also one of the countries with a high prevalence of stunting, where about 38% of children were stunted in 2016 [5]. The distribution of the problem also varies across different regions in Ethiopia where the highest (46%) prevalence of stunting reported in the Amhara region [5].

Women are often the primary caregivers for children, including food preparation and storage, feeding, psychosocial care, and hygienic practices [6]. Thus, their empowerment can influence the nutritional status of their children [6]. This is specially critical for child nutrition during the first 1000 days of life [7]. Although there is no standard and consistent measure of women’s empowerment among previous studies, in general, women’s empowerment refers to a process that involves the ability to take charge of their own lives such as the power and control over decisions and issues that shape their lives and the lives of their children [7, 8].

Studies have identified that women’s empowerment has a positive influence on child feeding practices and their growth and nutritional status [9–15]. For example, studies from Nepal [14], Bangladesh [9], Afghanistan [13], and India [15] revealed that mothers who have high empowerment were less likely to have stunted children. However, a study done in Brazil showed a positive association between women’s empowerment and having a stunted child [12]. A systematic review of literature has also shown an inconclusive relationship between women’s empowerment and child nutritional status [7]. Another evidence from sub-Saharan Africa (SSA) has revealed that the association of empowerment and its various dimensions with infant and young child feeding practices did not show any consistent and clear pattern across the countries studied [11].

The tools and/or dimensions used to define women’s empowerment are different in different contexts. For example, a study from Brazil [12] and some other studies from Southeast Asia [10, 15] focused solely on women’s autonomy and decision-making power as indicators of empowerment. Whereas a multi country study in SSA [11] used a bulk of other dimensions of empowerment such as economic, social, and legal aspects. Another study from Uganda measured decision-making and empowerment separately and revealed that both indicators were significantly positively associated with child feeding (minimally diverse and acceptable diet) [16]. Some researchers have also used the term ‘women’s status’ and defined it as the relative power of women compared to men interms of controlling household resources, accessing information and choosing healthcare services [17]. Generally, there is no universal definition for women’s empowerment, no consistent associations between empowerment and stunting, and quantifying the relationship between women’s empowerment and nutritional outcomes heavily relies on how empowerment and its dimensions are measured [7]. This suggests the need to consider context specific indicators of empowerment and to measure the independent effect of each dimension of women’s empowerment on child undernutrition.

Ethiopia is a developing country characterized by high gender inequality, with women marginalized in various socioeconomic aspects such as low levels of education, limited access to resources, economic dependence, low decision-making autonomy, and cultural suppression [18, 19]. These conditions may have contributed to the high prevalence of poor nutritional status among children in the country, especially in areas where childcare is solely women’s responsibilities in the community. Despite a range of socio-economic, demographic, and fertility related factors of stunting have been investigated in Ethiopia, little is known about women’s empowerment as one of the factors for stunting. Two previous studies from Ethiopia have shown that women’s empowerment leads to improvements in children’s dietary diversity [20, 21]. One of these studies further investigated the association between women’s empowerment, which was measured by women’s power to decide on major household expenses, and length-for-age but the association was not found to be significant [20]. Therefore, the aim of this study was to investigate whether women’s empowerment and its dimensions are associated with childhood stunting in the rural kebeles (lowest administrative units in Ethiopia) of Bahir Dar City Administration, northwest Ethiopia.

## Methods

### Study setting and period

A community-based cross-sectional study was conducted from February 10, 2019, to March 21, 2019, among currently married women of reproductive age who have children aged 6–59 months.

The study was conducted in three rural kebeles (Weramit, Wereb Qolatsion and Zenzelma) of Bahir Dar City Administration. Bahir Dar is the capital city of the Amhara Regional State, located 565 km northwest of Addis Ababa, the capital of Ethiopia. For administrative purposes, Bahir Dar is divided into nine sub cities, four satellite cities and nine rural kebeles. According to the City Administration Health Department report, the total population of the town in 2018 was estimated to be 339,683, of which 65,224 were living in rural kebeles. In the same year, the estimated number of under-five-year-old children was 46,007, with 8,834 of them were living in rural kebeles [22].

### Sample size and sampling procedure

The sample size was calculated using the single population proportion formula with the following assumptions: a 95% CI (z value of 1.96), 5% margin of error, and a 42.3% prevalence of stunting among children aged 6–59 months from a previous study in Gondar, northwest Ethiopia [23]. With an assumption of 10% non–response rate and taking design effect of 1.5 (for multistage systematic random sampling), the final sample size was 619.

To select study participants, multistage sampling technique was implemented. First, three kebeles were randomly selected from nine rural kebeles in Bahir Dar using a lottery method. Then, proportional allocation was used to determine the desired samples from each selected kebele. In the second stage, systematic random sampling was used to select eligible households, i.e., households with mothers having children aged 6–59 months. The sampling frame of all households with eligible under-five children was obtained from Health Extension Workers as they record all mothers and their children in their kebele under ‘the family folder’. Finally, eligible study participants were recruited, and a house-to-house data collection was conducted. If the mother had more than two eligible children, only the index (youngest) child was sampled to avoid the clustering effect and all the information was collected for the index child.

### Measurement and variables

The dependent variable was stunting, which was defined based on anthropometric measurments of height and age. The age of the child was based on the mothers response. To reduce recall bias, mothers response about their child’s age was crosschecked with immunization cards. In addition, respondents were asked to relate the childbirth date with major religious and cultural events. Height measurements were carried out using a Shorr measuring board (ShorrBoards®) and recorded to the nearest 0.1 cm. Children younger than 24 months were measured for length in recumbent position, and older children were measured while standing. Thus, stunting (height for age) was classified into a binary outcome variable (z-score below − 2 standard deviations as ‘stunted and ‘normal’ otherwise) based on the WHO growth standard and nutritional status classification criteria [2].

The main independent variables were the overall levels of women’s empowerment and its various dimensions. For this study, we identified five dimensions of women’s empowerment. The empowerment dimensions were identified based on a suggestion from a previous literature review to select women’s empowerment dimensions in context-specific ways [7] and through a review of literature from Ethiopia [20, 21] and globally [11, 14]. The five dimensions were household decision-making, educational status, cash earnings, house/land ownership, and membership in community groups. The five dimensions of empowerment and their categories are presented below (Table 1). The overall empowerment level was grouped into three categories as low (women who received a total score of two or less), moderate (a score of three or four), and high (a score of five or more), as previously suggested [14].

**Table 1:** Measures of women empowerment

Empowerment indicators	Score		
	0	1	2
Women’s involvement in household decision-making (access to health care, household purchasing, and freedom to visit relatives)	Did not participate in any decisions	Participated in one or two decisions	Participated in all three decisions
Women’s education	Did not attend school at all	Attended primary education	Attended secondary or higher education
Women’s cash earnings	Did not earn cash at all	Earned cash only or both cash and in-kind income	------------
Women’s ownership of house/land	Did not own a house, land, or both	Owned a house, land, or both either individually or jointly with husband	------------
Women’s membership in community groups	Was not a member of any community group	Was a member of community groups	------------

In addition, variables that needed to be controlled in order to estimate the unbiased independent association between the exposure and the outcome were identified from previous literature [9, 11–14, 20, 24–27]. Accordingly, variables related to mother’s characteristics (age, age at first marriage, household family size, access to media and employment status) and child factors (age, sex, birth order, breastfeeding status and morbidity status) were identified.

### Data collection tools and procedures

Socio-demographic data were collected using a structured questionnaire adapted from the 2016 Ethiopian Demographic and Health Survey [5]. The tool for measuring women’s empowerment was adapted from previous literature [7, 11, 14, 21, 28]. The anthropometric data were collected using the procedure stipulated by the WHO for taking anthropometric measurements [29]. The questionnaire was pre-tested on 30 participants in a similar setting (Addis Alem kebele) after being translated into the local Amharic language. However, there was no any difficulty in understanding the questions and no modifications were made. Trained data collectors with previous experience of anthropometric measures collected the data.

### Data processing and analysis

The collected data was coded and entered into Epi-data version 3.1. Anthropometric measurements were calculated using WHO Anthro software version 3.2.2. The data was analyzed using SPSS version 20.0. Binary logistic regression analyses were used to assess the association between the dependent variables with the main independent variable, as well as each control variables. Variables with a p-value of ≤ 0.2 in the binary logistic model were retained in the final multivariable logistic models to control potential confounding effects. Interactions between variables were assessed using variance inflation factor. Model fit was tested using Akaike Information Criteria and additional standard logistic regression diagnostic tests. Odds ratios, together with the 95%CI, were used to report the associations. Statistical significance was declared at a p-value of < 0.05.

## Results

### General characteristics of study participants

In this study, 582 mothers who have children aged 6–59 months participated, that gives a 94% response rate. The mean age of mothers was 28 years with standard deviation (SD ± 5.04) and the mean age of mothers at first marriage was 17 years (SD ± 4.07). Most (99%) of the mothers were Orthodox Christians and all participants were of Amhara ethnicity. About two-thirds of the mothers (77%) were housewives. The mean age of the children was 28 months (SD ± 13.93) and about half (51.5%) of the children were male (Table 2).

**Table 2:** Participant characteristics (n = 582)

Variable	Class		Frequency	%
Maternal age (years)	15–19		9	1.5
	20–24		121	20.8
	25–29		227	39.0
	30–34		139	23.9
	35–39		66	11.3
	40–49		20	3.4
	Mean ± SD		28.47 ± 5.04	
Maternal age at first marriage (years)	< 15		190	32.6
	15–18		96	16.5
	≥ 18		296	50.9
	Mean ± SD		17.27 ± 4.07	
Religion	Orthodox Christian		576	99.0
	Muslim		6	1.0
Occupation	Housewife		448	77.0
	Employed		15	2.6
	Merchant		25	4.3
	Farmer		70	12.0
	Daily labourer		24	4.1
Total family size	Median, Range		4, (3–9)	
Number of under five children	1		519	89.2
	≥ 2		63	10.8
Media (radio/TV) exposure	Yes		40	6.9
	No		542	93.1
Age of child (months)	6–11		67	11.5
	12–23		163	28.0
	24–35		176	30.2
	36–47		112	19.2
	48–59		64	11.0
	Mean age ± SD		28.25 ± 13.93	
Sex of child	Male		300	51.5
	Female		282	48.5
Birth order	1st		223	38.3
	2nd		158	27.1
	3rd or higher		201	34.5
Had illness*	Yes		56	9.6
	No		526	90.4
Breastfeeding status	Still breast feeding	Age (months)		
		6–11	64	15.8
		12–23	154	37.9
		24–35	136	33.5
		36–47	44	10.8
		48–59	8	2.0
	Stop breast feeding	6–11	3	1.7
		12–23	9	5.1
		24–35	40	22.7
		36–47	68	38.6
		48–59	56	31.8

SD, standard deviation

*Had diarrhoea, fever, cough in the last two weeks before the survey

### Prevalence of stunting in children

**Fig. 1:**
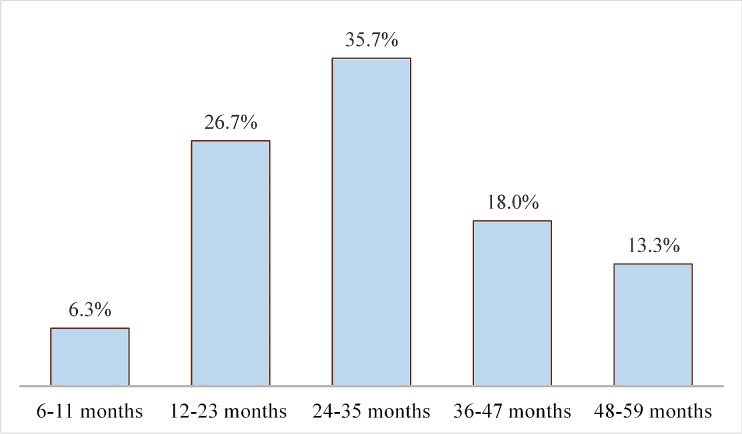
Proportion of stunted children by age group

In the current study, the prevalence of stunting among children aged 6–59 months was 255 (43.8%). The mean (± SD) of height-for-age score was − 1.77 ± 1.45 (95% CI: -1.89, -1.65). The prevalence of stunting was higher among female children; 158 (62.0%) of all stunted children were females. The prevalence of stunting among female children was 55.3% while the prevalence of stunting among male children was 33.0%. The nutritional status of children, categorized by age, showed that the 24–35 months had the highest (35.7%) prevalence of stunting (Fig. 1).

### Women’s empowerment dimensions

Table 3 shows the overall women’s empowerment level and its dimensions. More than half (54%) of the mothers were moderately empowered. About 19% of mothers had participated in all three household decision-makings processes, 52% had received formal education, 29% were members of community groups, 30% had cash earnings, and 70% owned a house or land (Table 3).

**Table 3:** Magnitude of women’s empowerment and its dimensions (n = 582)

Variable		Class	Number	%
Mothers’ empowerment level		Low	114	19.6
		Moderate	312	53.6
		High	156	26.8
Mothers’ empowerment dimensions	Household decision-making	No participation	43	7.4
		Participate in one or two decisions	110	18.9
		Participate in all three decisions	429	73.7
	Educational status	No education	282	48.5
		Primary level education	196	33.7
		Secondary and above	104	17.9
	Cash earnings	Yes	175	30.1
		No	407	69.9
	House/land ownership	Yes	406	69.8
		No	176	30.2
	Membership in community groups	Yes	166	28.5
		No	416	71.5

### The association between women’s empowerment and stunting

In the final adjusted logistic regression model, there was a significant association between mothers’ empowerment and stunting in their children. Accordingly, the odds of having stunted children was 40% lower (AOR 0.60, 95% CI: 0.35, 0.97) among mothers with moderate empowerment compared to mothers with low empowerment. Likewise, the odds of having stunted children was 44% lower (AOR 0.56, 95% CI: 0.37, 0.86) among mothers with high empowerment than mothers with low empowerment. Regarding each empowerment dimension, mothers who completed secondary education or higher were 43% (AOR 0.57, 95% CI: 0.35, 0.93) less likely to have a stunted child compared to mothers with no formal education. However, there was no significant association between primary level of education and stunting (AOR 1.29, 95% CI: 0.87, 1.92). Compared to women who had no house or land ownership, those who owned a house or land were less likely to have a stunted child (AOR 0.64, 95% CI: 0.44, 0.94). Likewise, mothers who were members of community groups were 46% (AOR 0.54, 95% CI: 0.36, 0.80) less likely to have a stunted child compared to mothers who were not members of community groups. Two of the empowerment dimensions (household decision-making and cash earning) did not show a significant association with stunting in the final model (Table 4).

**Table 4:** Associations between women’s empowerment and stunting

Variable	Class	Stunting			
		COR (95% CI)	p	AOR (95% CI)^a^	p
Empowerment level	Low	Reference			
	Moderate	0.74 (0.45, 1.19)	0.214	0.60 (0.35, 0.97)	0.045
	High	0.67 (0.46, 0.99)	0.042	0.56 (0.37, 0.86)	0.008
Household decision-making	No participation	1.14 (0.61, 2.13)	0.693	1.26 (0.66, 2.41)	0.485
	Participate in 1–2	Reference			
	Participate in all	0.63 (0.41, 0.71)	0.036	0.81 (0.52, 1.29)	0.378
Educational status	No formal education	Reference			
	Primary	0.99 (0.68, 1.42)	0.935	1.29 (0.87, 1.92)	0.206
	Secondary+	0.51 (0.31, 0.81)	0.005	0.57 (0.35, 0.93)	0.025
Cash earnings	No	Reference			
	Yes	0.56 (0.39, 0.81)	0.002	0.76 (0.51, 1.13)	0.174
House or land ownership	No	Reference			
	Yes	0.58 (0.40, 0.84)	0.004	0.64 (0.44, 0.94)	0.024
Membership in community groups	No	Reference			
	Yes	0.50 (0.35, 0.72)	< 0.001	0.54 (0.36, 0.80)	0.002

COR, crude odds ratio; CI, confidence interval; AOR, adjusted odds ratio

^a^ The model was adjusted for mother’s occupational status, family size, media exposure, child sex, child age, and child birth order; p, p-value

## Discussion

This study assessed women’s empowerment (including its individual dimensions) and its association with childhood stunting. The results revealed that three of women’s empowerment dimensions (education, land ownership, and membership in community groups) were significantly associated with stunting in children even after controlling for potentially confounding variables. Hence, efforts to enhance women’s education, ensure their land ownership (through access to credit or policy interventions), and improve their involvement in local community groups are highly likely to improve the nutritional status of their children.

In this study, mothers who attended secondary education or above were less likely to have stunted children compared to mothers who did not attend any education, which is consistent with previous research [9, 20, 30, 31]. Higher education enables women to make independent choices, be accepted by other household members, contribute to a change in household income, and have greater access to household resources [32], which are crucial for improving the nutritional status of children. High educational attainment can also lead to improvements in women’s cognitive skills, which can in turn impact their health knowledge, household income, and consequently, the health and nutritional welbeing of their children [33, 34]. The results suggest that in our study area, where education among girls remains unacceptably low and cultural marriage patterns continue to place mothers in vulnerable social positions, tailored educational policies to ensure women’s completion of their secondary education are essential for improving child nutrition.

Our findings also showed that mothers who owned a house or land were less likely to have stunted children compared to their counterparts. This empowerment domain reflects women’s ability to access and control resources [7], making it a more appropriate proxy measure in our study context where our respondents were rural communities. Women’s control over household or land helps to enhance their economic security and strengthen their control over resources which contributes to maintaining household food security and food diversity [7], ultimately improving child nutrition.

Being a member of community groups was associated with a lower likelihood of having a stunted child. Another study from Ethiopia also indicated that women’s participation in group membership has a positive and significant impact on dietary diversity and improved nutritional outcomes for children [21]. Women’s groups assist in creating a supportive environment where women can express their opinions and share experiences. This can build their confidence, gain community support, and be able to make informed decisions that promote their own health and that of their children. Therefore, connecting women within their own village to promote behavioral change communication and information sharing might improve child nutrition. Such interventions could promote breastfeeding practices, dietary frequency and diversity, hygienic practices during food preparation and feeding, and childcare services including growth monitoring and immunization.

Contrary to the general perception, women’s participation in household decision-making was not significantly associated with stunting. This finding is consistent with previous studies [12, 20, 35] but contradicts other studies [9, 10, 13, 15]. A previous multi-country study from east Africa (including Ethiopia) revealed that women’s decision-making was not significantly associated with stunting [35]. There is an argument on the effect of women’s participation in decision-making on child nutritional status. Some scholars argue that women’s active participation in domestic decision-making reflects their power within the household and may increase their likelihood of making informed choices. For example, women’s decision on household purchases increases their ability to choose what food to buy and how much to buy; their decision-making power in healthcare improves the quality of childcare; and their decision-making autonomy to visit family reflects their access to information [28]. However, some argue that the relative power and control over resources in some households may not have a significant effect on women’s wellbeing and on the state of their children. For example, in certain households, where both men and women work together and resources are pooled to improve the wellbeing of the household, women’s decision-making role may not impact health in the same way [21]. We suggest further studies to investigate the mechanisms through which women’s autonomy might affect the nutritional status of their children.

We hypothesized that in communities where women are the primary housekeepers and caregivers of children, cash earned and/or controlled by women is more likely to be used to purchase food and healthcare for children and thereby improving child nutrition. However, our findings did not support this theory, as no significant association was observed between cash earnings and stunting. The time women spend in the workplace could reduce the amount of time available for them to take care of themselves and their children, which might partly explain the lack of association regarding the impact of cash earning on child nutrition [21].

Our findings should be interpreted considering some limitations. First, this cross-sectional study is unable to establish a cause-and-effect relationship. This may particularly occur when women with stunted children dedicate their time to caring for their malnourished child at the expense of time to spend on empowerment activities such as education and community participation. Second, the absence of a significant relationship between decision-making autonomy and stunting could be affected by endogeneity bias i.e., decision-making variable could be changed or determined by its relationship with other variables within the model such as women’s education and group membership. Third, some women may not accurately remember the age of their child, which may lead to recall bias.

## Conclusion

This study revealed that overall women’s empowerment and some of its dimensions (secondary and above education, house or land ownership, and membership in community groups) are associated with lower likelihood of childhood stunting. The findings suggest the need to design and integrate women empowerment strategies into child nutrition programs.

## Data Availability

The datasets generated and/or analysed during the current study are not publicly available due to participants’ consent were not obtained to share their data to third party but are available from the corresponding author on reasonable request.

## Abbreviations

AOR: Adjusted odds ratio
CI: Confidence interval
COR: Crude odds ratio
SD: Standard deviation
SSA: Sub-Saharan Africa
WHO: World Health Organisation
